# Ischemia-Related Subcellular Redistribution of Sodium Channels Enhances the Proarrhythmic Effect of Class I Antiarrhythmic Drugs: A Simulation Study

**DOI:** 10.1371/journal.pone.0109271

**Published:** 2014-10-03

**Authors:** Kunichika Tsumoto, Takashi Ashihara, Ryo Haraguchi, Kazuo Nakazawa, Yoshihisa Kurachi

**Affiliations:** 1 Department of Pharmacology, Graduate school of Medicine, Osaka University, Suita, Japan; 2 Department of Cardiovascular Medicine, Shiga University of Medical Science, Otsu, Japan; 3 Department of Medical Informatics, National Cerebral and Cardiovascular Center, Suita, Japan; 4 Laboratory of Biomedical Science and Information Management, Research Institute, National Cerebral and Cardiovascular Center, Suita, Japan; 5 Center for Advanced Medical Engineering and Informatics, Osaka University, Suita, Japan; University of Minnesota, United States of America

## Abstract

**Background:**

Cardiomyocytes located at the ischemic border zone of infarcted ventricle are accompanied by redistribution of gap junctions, which mediate electrical transmission between cardiomyocytes. This ischemic border zone provides an arrhythmogenic substrate. It was also shown that sodium (Na^+^) channels are redistributed within myocytes located in the ischemic border zone. However, the roles of the subcellular redistribution of Na^+^ channels in the arrhythmogenicity under ischemia remain unclear.

**Methods:**

Computer simulations of excitation conduction were performed in a myofiber model incorporating both subcellular Na^+^ channel redistribution and the electric field mechanism, taking into account the intercellular cleft potentials.

**Results:**

We found in the myofiber model that the subcellular redistribution of the Na^+^ channels under myocardial ischemia, decreasing in Na^+^ channel expression of the lateral cell membrane of each myocyte, decreased the tissue excitability, resulting in conduction slowing even without any ischemia-related electrophysiological change. The conventional model (i.e., without the electric field mechanism) did not reproduce the conduction slowing caused by the subcellular Na^+^ channel redistribution. Furthermore, Na^+^ channel blockade with the coexistence of a non-ischemic zone with an ischemic border zone expanded the vulnerable period for reentrant tachyarrhythmias compared to the model without the ischemic border zone. Na^+^ channel blockade tended to cause unidirectional conduction block at sites near the ischemic border zone. Thus, such a unidirectional conduction block induced by a premature stimulus at sites near the ischemic border zone is associated with the initiation of reentrant tachyarrhythmias.

**Conclusions:**

Proarrhythmia of Na^+^ channel blockade in patients with old myocardial infarction might be partly attributable to the ischemia-related subcellular Na^+^ channel redistribution.

## Introduction

Class I antiarrhythmic drugs, which block cardiac sodium (Na^+^) channels, have been used to treat premature ventricular contractions (PVCs), which degenerate into tachyarrhythmias. The Cardiac Arrhythmia Suppression Trial (CAST) [1], [2] showed that the risk of arrhythmia-related death was increased in patients with old myocardial infarction, although Na^+^ channel blockers reduced PVCs. Therefore, the administration of Na^+^ channel blockers to old myocardial infarction patients is currently contraindicated. However, it remains controversial whether the poor prognosis is due to the negative inotropic and/or proarrhythmic effects of Na^+^ channel blockers.

Previous experimental studies [3]–[9] showed that electrophysiological remodeling occurs in cardiomyocytes located at the ischemic border zone (IBZ) of infarcted ventricles. In particular, functional remodeling [3], [4] of Na^+^ channel current (*I*_Na_), i.e., reduction in the current amplitude and alterations in the activation and/or inactivation kinetics, was observed in myocytes isolated from the IBZ, consequently decreasing the conduction velocity (CV) of the excitation wavefront. If the IBZ exhibits such *I*_Na_ remodeling, it might become a substrate of ventricular tachyarrhythmias.

The Na^+^ channels of the mammalian heart were inhomogeneously distributed within myocytes, particularly at intercalated discs (IDs) [10]–[13]. Baba *et al*. [14] found that the subcellular redistribution of Na^+^ channels occurs at the IBZ of 5-day infarcted canine ventricles. In addition, the Na^+^ channel expressions in the lateral cell membrane were markedly decreased, and thus the Na^+^ channels were confined to the IDs. However, the roles of the subcellular redistribution of Na^+^ channels in the proarrhythmic effects of Na^+^ channel blockers in addition to ventricular tachyarrhythmias under ischemia remain unclear.

A hypothetical conduction mechanism based on the microstructure of ventricular myocytes, i.e., the electric field (EF) mechanism, has initially proposed as the main mechanism of excitation conduction [15]. However, it has been theoretically confirmed that the contribution of the gap junction mechanism is larger than that of the EF mechanism in the physiological condition [12] and the EF mechanism serves as one of the homeostatic mechanisms of excitation conduction under conditions of reduced gap junctional coupling in the diseased heart [16], [17]. At the same time we found that because of the EF mechanism, CV is very sensitive to the amount of Na^+^ channels of lateral surface membrane (LM) despite normal gap junctional coupling [16]. In the present study, we extend this idea to the proarrhythmic mechanism under the conditions of 5-day infarcted canine heart, of which gap junctional coupling is not reduced yet [8], [9]. The aim of this study was to investigate the combined effects of the subcellular redistribution of Na^+^ channels and the Na^+^ channel blockade on excitation conduction. The roles of the subcellular redistribution of Na^+^ channels in excitation conduction were investigated by altering the subcellular Na^+^ channel distribution of each myocyte in a myofiber model. In addition, the proarrhythmic effects of class I antiarrhythmic drugs on infarcted ventricular tissue were evaluated on the basis of the conduction properties in the simulated myofibers.

## Methods

### Myofiber model

**Figure 1:**
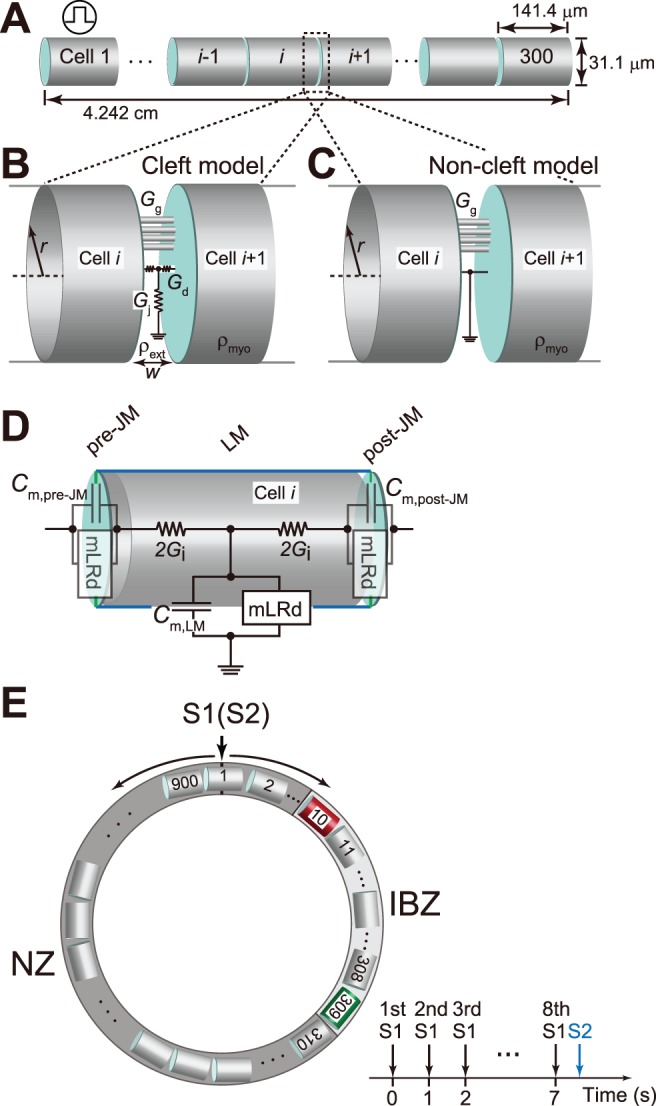
Cleft and non-cleft models of myocardial fibers and rings. (A), Schematic representation of a myocardial fiber comprising cylindrical 300 cells. (B) and (C), Schematic representations of the intercellular junction in the cleft (B) and non-cleft (C) models. (D), The AP of each membrane segment is represented by the modified Luo–Rudy dynamic (mLRd) ventricular myocyte model [24], [25]. (E), Schematic representation of the myocardial ring comprising 900 cells (a), and the pacing protocol (b). *G*_g_, gap junctional conductance; *G*_j_, radial cleft conductance; *G*_d_, axial cleft conductance.

We constructed a myofiber of length 4.242 cm, consisting of 300 ventricular myocytes, each of which was 141.4 µm in length and 31.1 µm in width [18]–[20] (Figure 1A). The myocytes were electrically connected with both gap junctions and the EF mechanism; the electrical communication between the adjacent cells was mediated by the change in the large negative extracellular potential induced at the narrow intercellular cleft space facing the junctional membrane (JM), i.e., the ID (cleft model [12], [15]–[17], Figures 1B and S1). For comparison with the cleft model, a conventional model [21] coupled only with gap junctions was constructed (non-cleft model, Figure 1C). On the basis of previous experimental data [8], [9] showing that the intercellular gap junctional conductance (*G*_g_) of the IBZ in 5-day infarcted canine heart does not differ significantly from that of the non-ischemic zone (NZ), we employed the same *G*_g_ in both the IBZ and NZ myofiber models: 3.584 µS. The radial cleft conductance (*G*_j_) [22] and series axial cleft conductance (*G*_d_) were 0.25 µS and 33.8 mS, respectively, the same as in previous studies [12], [15]–[17].

Each myocyte in the myofiber model comprised 3 segments: 2 for the JM (i.e., post- and pre-JMs), and the other one for the LM (Figure 1D). The membrane segments were represented by a modified Luo–Rudy dynamic (mLRd) model [16], [23]–[26]. Details of the myofiber and the mLRd model are provided in the Text S1.

### Myocardial ring model

To evaluate the effects of Na^+^ channel blockade on the vulnerability of a myocardial ring model comprising an NZ and IBZ (Figure 1E, left) to PVCs, additional simulations were performed using the S1–S2 stimulation protocol: 8 S1 stimuli with a basic cycle length of 1,000 ms were applied transmembranously followed by an S2 stimulus with various coupling intervals (Figure 1E, right). The diameter of the myocardial ring model was 4.0 cm. There were 300 (cells 11–310) and 600 (cells 311–10) cells in the IBZ and NZ, respectively.

### Subcellular Na^+^ channel distribution and Na^+^ channel blockade

The Na^+^ channel conductance was 16 mS/cm^2^ [24]. Thus, the entire Na^+^ channel conductance of each myocyte corresponded to 2.45 µS (*G*_Na_), which was defined as the control value. We altered the subcellular distribution of Na^+^ channels by allocating the entire Na^+^ channel conductance to each membrane segment. The Na^+^ channel conductance of the JM and LM are expressed as percentages of the *G*_Na_: %*g*_Na,JM_ and %*g*_Na,LM_, respectively. Thus, the entire Na^+^ channel conductance (%*g*_Na,JM+LM_) was equal to the sum of %*g*_Na,JM_ and %*g*_Na,LM_.

As the *I*_Na_ amplitude at the LM becomes about the same as that of IDs [27], [28], we determined the subcellular Na^+^ channel distribution in the NZ. In particular, the Na^+^ channel conductance in the JM and LM of a myocyte located in the NZ were estimated to be half of the entire Na channel conductance, i.e., 50%*g*_Na,JM_ and 50%*g*_Na,LM_ [27]–[29]. Meanwhile, the subcellular Na^+^ channel distribution in the IBZ was defined as the distribution to reproduce the experimentally measured CV [9] referring to previous experimental observations [14] that the Na^+^ channels are selectively decreased from the LM.

Na^+^ channel blockade by the administration of class I antiarrhythmic drugs was achieved by reducing the entire Na^+^ channel conductance while maintaining the ratio of %*g*_Na,JM_ to %*g*_Na,LM_. The ratio of Na^+^ channel blockade is expressed as a percentage of the *G*_Na_ (%*G*_Na_ block). For instance, 30%*G*_Na_ block with 50%*g*_Na,JM_ and 50%*g*_Na,LM_ (%*g*_Na,JM_/%*g*_Na,LM_ = 1) indicates that Na^+^ channels were reduced to 35%*g*_Na,JM_ and 35%*g*_Na,LM_.

### Computations

Numerical calculations were performed as described previously [16] and details are provided in Text S1. Pacing stimuli of twice the diastolic threshold were applied to the LM segment of a myocyte located at one end of the myofiber. To obtain the CV restitution curves, we measure the CV in the middle of the myofiber as a function of the S1–S2 coupling interval.

## Results

### Effects of subcellular Na^+^ channel redistribution on CV

**Figure 2:**
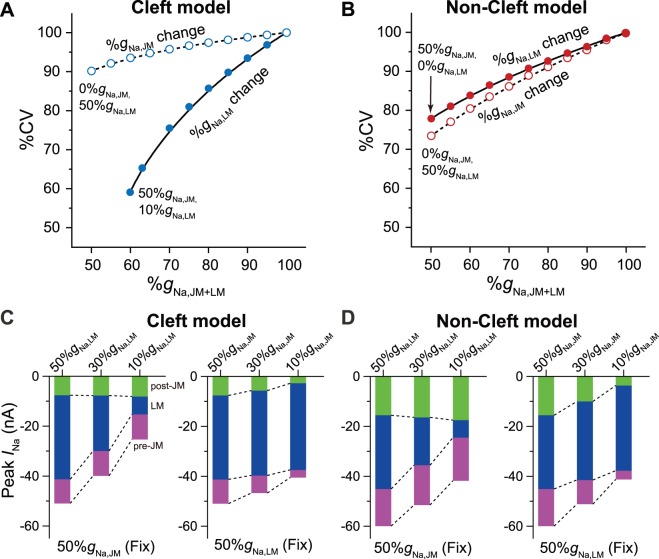
Conduction velocities and changes in regional *I*_Na_ in cardiomyocytes in each myofiber model. (A) and (B), Relative ratios of CV (%CV) normalized by CV in the myocardial fiber with 50%*g*_Na,JM_ and 50%*g*_Na,LM_ as a function of %*g*_Na,JM_ or %*g*_Na,LM_ in the cleft (A) and non-cleft (B) models. (C), Peak values of post-junctional *I*_Na_ (post-*I*_Na,JM_), pre-junctional *I*_Na_ (pre-*I*_Na,JM_), and lateral *I*_Na_ (*I*_Na,LM_) in a myocyte in the cleft model in which the Na^+^ channels are fixed at 50%*g*_Na,JM_ with 50%*g*_Na,LM_, 30%*g*_Na,LM_, or 10%*g*_Na,LM_ (*left*) and at 50%*g*_Na,LM_ with 50%*g*_Na,JM_, 30%*g*_Na,JM_, or 10%*g*_Na,JM_ (*right*). (D), Peak values of post-*I*_Na,JM_, pre-*I*_Na,JM_, and *I*_Na,LM_ in a myocyte in the non-cleft model with 50%*g*_Na,JM_ and 50%*g*_Na,LM_, 30%*g*_Na,LM_, or 10%*g*_Na,LM_ (*left*), and 50%*g*_Na,LM_ with 50%*g*_Na,JM_, 30%*g*_Na,JM_, or 10%*g*_Na,JM_ (*right*). The peak values of *I*_Na_ in each membrane segment were measured in the middle of the myofiber.

Figures 2A and 2B show the relative ratios of CV (%CV) normalized by CV in the myocardial fiber with 50%*g*_Na,JM_ and 50%*g*_Na,LM_, i.e., the subcellular Na^+^ channel distribution in the NZ, as a function of %*g*_Na,JM+LM_ in the cleft and non-cleft models, respectively. Thus, the CVs in the cleft and non-cleft models under NZ myofiber condition were 53.1 and 64.3 cm/s, respectively. In the case of %*g*_Na,JM_ change (Figure 2A, open circles with dashed line), the %CV decreased not more than 10% as the %*g*_Na,JM_ was reduced under the condition with 50%*g*_Na,LM_. Meanwhile, in the case of %*g*_Na,LM_ change (Figure 2A, filled circles with solid line), the %CV decreased markedly as a function of %*g*_Na,LM_ decrease under the condition with 50%*g*_Na,JM_. However, in the non-cleft model (Figure 2B), there was no significant difference in the change in %CV in either case.

On the basis of the experimentally measured CV [9] with reference to the previous immunostaining data [14], we determined the subcellular Na^+^ channel distribution in the IBZ as 50%*g*_Na,JM_ and 15%*g*_Na,LM_; the CVs in the cleft and non-cleft models were 36.4 and 55.7 cm/s, respectively.

### Contributions of *I*_Na_ in each segment to CV

Next, we investigated the contributions of both %*g*_Na,JM_ and %*g*_Na,LM_ in each membrane segment to the total *I*_Na_, which was defined as the sum of post-junctional, pre-junctional, and lateral *I*_Na_ values of each myocyte in the myofiber (Figures 2C and 2D).

When %*g*_Na,JM_ was fixed at 50% in the cleft model, the peak value of *I*_Na_ in the LM (Figure 2C, *left*, blue bars) decreased gradually as a result of the reduction in %*g*_Na,LM_. The total peak *I*_Na_ per myocyte (i.e., the maximum value of each bar in Figure 2C, *left*) also decreased substantially as a result of the reduction in %*g*_Na,LM_. In contrast, if %*g*_Na,JM_ was reduced with 50%*g*_Na,LM_, the peak value of *I*_Na_ in the JM (Figure 2C, *right*, green and magenta bars) also decreased. However, the decrease in the total peak *I*_Na_ (i.e., maximum value of each bar in Figure 2C, *right*) only decreased slightly, suggesting the *I*_Na_ in the JM (*I*_Na,JM_) contributes less to the total peak *I*_Na_ than the *I*_Na_ in the LM (*I*_Na,LM_).

For comparison, similar simulations were performed in the non-cleft model (Figure 2D). The *I*_Na,LM_ decreased along with the reduction in %*g*_Na,LM_ (Figure 2D, *left*, blue bars), consequently decreasing the total peak value of the *I*_Na_ (i.e., maximum value of each bar in Figure 2D, *left*). Likewise, *I*_Na,JM_ decreased along with the reduction in the %*g*_Na,JM_ (Figure 2D, *right*, green and magenta bars); the total peak value of the *I*_Na_ also decreased (i.e., maximum value of each bar in Figure 2D, *right*). However, there was no striking difference in the change in the total peak value of *I*_Na_ in either case (Figures 2D, *left* and *right*).

### Effects of Na^+^ channel blockade on CV in NZ and IBZ myofibers

**Figure 3:**
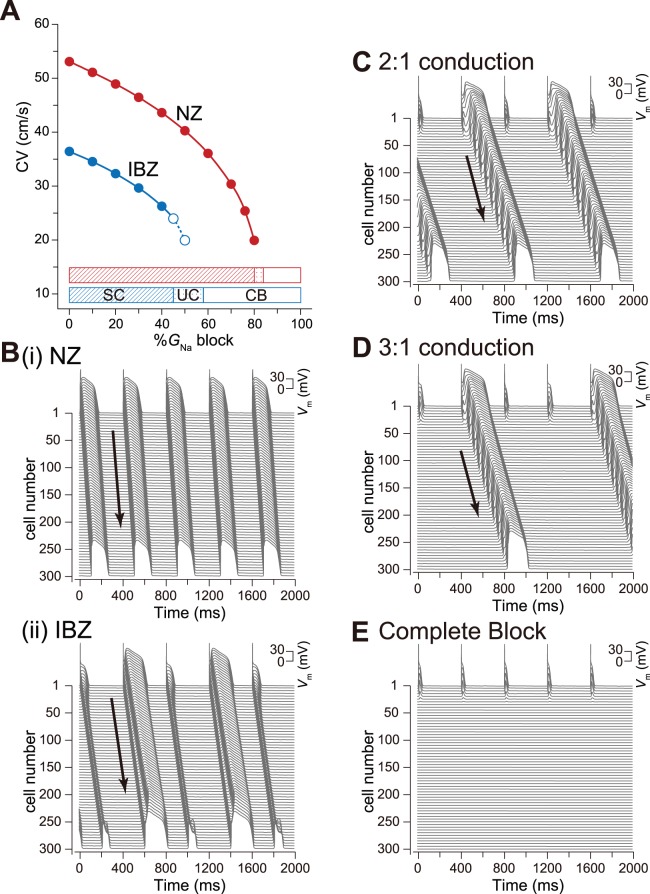
Destabilization of action potential propagation by Na^+^ channel blockade. (A), Conduction velocity (CV) as a function of %*G*_Na_ block. Open circles with the dashed lines denote CV under AP alternans. The relationship between the excitation conduction mode and %*G*_Na_ block is represented by the bottom bars. SC, stable conduction; UC, unstable conduction (e.g., AP alternans, or 2∶1 or 3∶1 conduction); CB, complete conduction block. (B), Examples of AP propagation observed in NZ (i) and IBZ (ii) myofibers. (C)–(E), Examples of 2∶1 conduction by 55%*G*_Na_ block (C), 3∶1 conduction by 56% *G*_Na_ block (D), and complete conduction block by 60% *G*_Na_ block (E) in the IBZ.

Figure 3A shows CV slowing as a function of %*G*_Na_ block. In both NZ and IBZ myofibers, CVs decreased gradually with %*G*_Na_ block. Figure 3B shows typical examples of the AP propagation observed in NZ and IBZ myofibers with 50%*G*_Na_ block. The AP of each myocyte in the NZ myofiber with 50%*G*_Na_ block was able to propagate through the myofiber (Figure 3B(i)), whereas the same 50%*G*_Na_ block in the IBZ myofiber caused AP alternans (Figure 3B(ii)). The ionic mechanism underlying AP alternans is shown in Figure S3. The reduction in *I*_Na,LM_ by the Na^+^ channel blocker decreased in the AP phase 0 amplitude (Figures S3A and S3B, asterisk), leading to slow activation of L-type Ca^2+^ current, *I*_CaL_ (Figure S3C). The slow *I*_CaL_ activation prolonged AP duration in the IBZ myocytes, delaying phase 2 dome formation in the AP (Figure S3A). The longer AP duration, i.e., the diastolic interval (DI) shortening, further decreased *I*_Na,LM_ (Figure S3B, dagger). Consequently, the AP phase 0 amplitude decreased further, and the *I*_CaL_ failed to activate (Figures S3A and S3C, dagger). The failure of *I*_CaL_ activation led to the shortening of AP duration and prolongation of the DI. The peak *I*_Na,LM_ recovered slightly as a result of the prolongation of the DI, eliciting the full AP accompanied by the delayed phase 2 dome. The critical values of the %*G*_Na_ block maintaining stable conduction were 80% and 44% in the NZ and IBZ myofibers, respectively (Figure 3A, bottom, red and blue bars denoted by SC, respectively). Increases in %*G*_Na_ block exceeding the critical value in both NZ and IBZ myofibers caused unstable conduction. Typical examples of unstable conduction observed in the IBZ myofiber are shown in Figures 3C and 3D. The further increase in %*G*_Na_ block caused complete conduction block (Figure 3E). In the NZ myofiber, the AP did not propagate when the entire Na^+^ channel conductance within the myocyte was reduced by 84%. Complete conduction block occurred in the IBZ myofiber with a %*G*_Na_ of 58%.

### CV restitution properties

**Figure 4:**
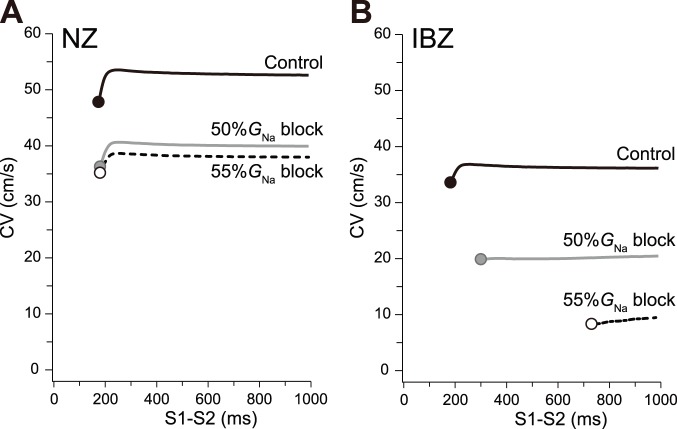
Conduction velocity restitution properties. Conduction velocity (CV) restitution curves in the NZ (A) and IBZ (B) myofiber models as a function of S1–S2 interval. Ten S1 stimuli of basic cycle length (400 ms) were applied transmembranously followed by an S2 stimulus with various coupling intervals.

CV as a function of S1–S2 interval is shown in Figure 4. In the case of the NZ myofiber (Figure 4A), although the CV with 50%*G*_Na_ block was markedly lower than that of the control, the minimum S1–S2 interval resulting in conduction block did not change (178 vs. 171 ms in the control, filled and gray circles at left end of each curve, respectively). Meanwhile, in the case of the IBZ myofiber (Figure 4B), the conduction block occurred when the S1–S2 interval was <181 and <298 ms under the control and 50%*G*_Na_ block, respectively. Further increasing the %*G*_Na_ block resulted in the conduction block occurring with an S1–S2 interval of 730 ms (Figure 4B, open circle at the left end of 55%*G*_Na_ block curve). Thus, the S1–S2 interval causing conduction block was markedly prolonged with increasing %*G*_Na_ block of IBZ myocytes. However, in NZ myocytes, even if the %*G*_Na_ block was increased by 55%, the S1–S2 interval resulting in conduction block was only 179 ms (Figure 4A, open circle).

### Reentry induction in a myocardial ring model

**Figure 5:**
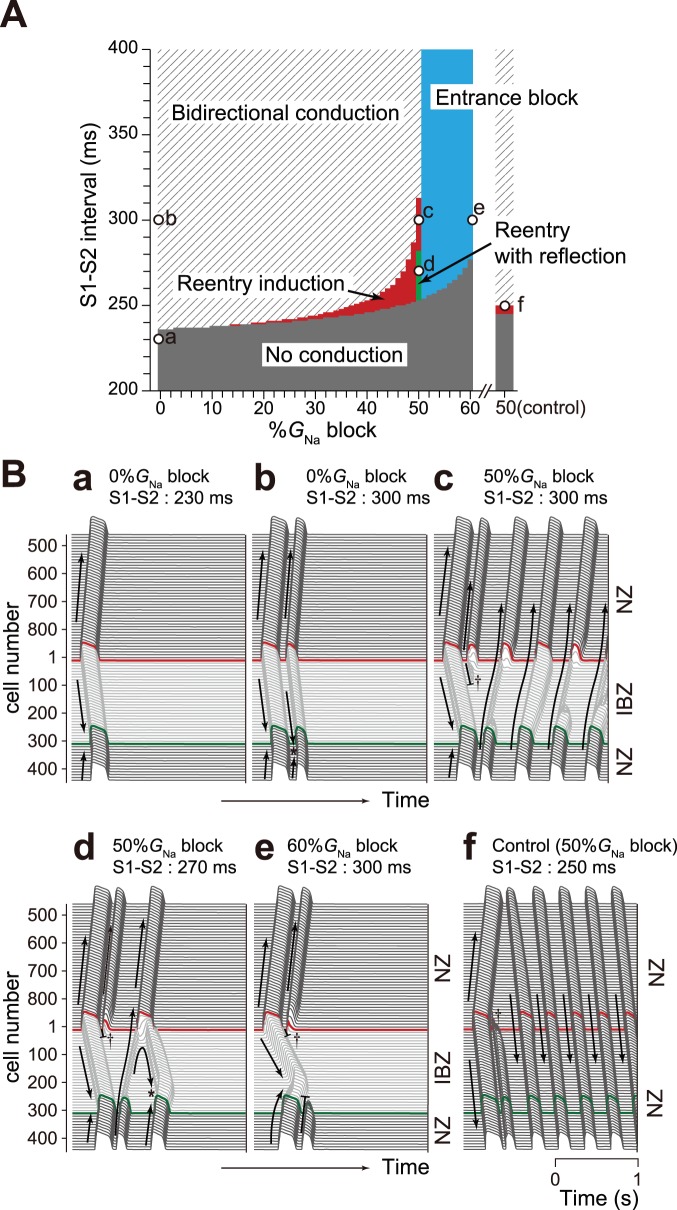
Reentry induction by Na^+^ channel blockade in myocardial ring models. (A), Phase diagram of %*G*_Na_ block vs. S1–S2 interval showing proarrhythmic events under Na^+^ channel blockade in the myocardial ring model. Right bar (control): responses to S2 stimulus in the myocardial ring constructed of only NZ myocytes under 50%*G*_Na_ block. (B), Examples of AP propagation in response to S1–S2 interval. Arrows and black short bars indicate the directions of AP propagation and entrance block, respectively.

A phase diagram of the excitation conduction in response to S1–S2 interval for a given degree of Na^+^ channel blockade is shown in Figure 5A. Open circles labeled a–f in Figure 5A correspond to Figures 5B(a)–(f), respectively. The gray region in Figure 5A represents the failure of AP induction due to the refractory period at the S2 stimulus site (Figure 5B(a)). Meanwhile, the shaded region in Figure 5A denotes the bidirectional conduction from the S2 stimulus site, resulting in the collision of excitation waves (Figure 5B(b), asterisk). The red region in Figure 5A represents the conditions of reentry (i.e., counterclockwise rotation) induction shown in Figure 5B(c). The green region in Figure 5A (i.e., the S1–S2 intervals from 254–280 ms in the case of 50%*G*_Na_ block) led to reentry via unidirectional conduction block at the proximal border of the IBZ (Figure 5B(d), dagger) followed by spontaneous termination with a collision between the counterclockwise-rotating wavefront and the reflection [30] (phase 2 reentry [31])-mediated clockwise rotating wavefront (Figure 5B(d), asterisk). Similar to the AP alternans, the *I*_Na,LM_ in the IBZ myocytes diminished by Na^+^ channel blockers led to a reduced AP phase 0 amplitude, failed *I*_CaL_ activation, and abbreviated AP (Figures S4A, S4C, and S4D). When the excitation wavefront reached the border with the NZ region, NZ myocytes were able to generate a full AP because they possessed the higher tissue excitability of the NZ compared to the IBZ (Figure S4A). The larger difference in potential between the NZ and IBZ myocytes subsequently elicited the gap junctional current from NZ to IBZ myocytes (Figure S4B), causing the reactivation of *I*_CaL_ in IBZ myocyte (Figure S4D, asterisk). The reactivated *I*_CaL_ depolarized the membrane potential of IBZ myocytes again (Figure S4A, asterisk), resulting in the generation of the reflection. The blue region in Figure 5A (i.e., further increase in the %*G*_Na_ block) denotes the conduction block occurring at both the proximal and distal borders of the IBZ (Figure 5B(e)). In contrast, in the case of 50%*G*_Na_ block in the control model without an IBZ, the S1–S2 intervals initiating reentry (Figure 5B(f)) were markedly narrower (Figure 5A, red region of the right bar).

## Discussion

The major findings of the present study are as follows: (1) a decrease in the number of Na^+^ channels from the LM of each ventricular myocyte was a major cause of the conduction slowing in the IBZ; (2) in the presence of EF mechanism, the relative contribution of the *I*_Na,LM_ to the total *I*_Na_ in a myocyte that makes up myofibers was greater than that of *I*_Na,JM_; (3) an IBZ accompanied by the subcellular redistribution of Na^+^ channels was highly vulnerable to reentry under Na^+^ channel blockade. These findings suggest that the subcellular Na^+^ channel redistribution in the IBZ leads to decreased tissue excitability and that such a phenomenon is facilitated by Na^+^ channel blockers. Na^+^ channel blockade tended to cause a unidirectional conduction block toward the IBZ, resulting in the induction of reentrant tachyarrhythmia following PVC. Thus, the ischemia-related subcellular redistribution of Na^+^ channels might be partly responsible for the proarrhythmic effects of Na^+^ channel blockers in patients with old myocardial infarction.

### Conduction slowing caused by ischemia-related subcellular Na^+^ channel redistribution

Cabo *et al*. [9] found that the CV in the IBZ of 5-day infarcted canine ventricles is 36% slower than that in the NZ (29 vs. 45 cm/s, respectively). The experimentally observed conduction slowing in the IBZ [9] may be attributable to the subcellular Na^+^ channel redistribution via EF mechanism, because the decreased number of Na^+^ channels in the LM of myocytes in the cleft model resulted in a similar decrease in CV (31.3%; Figure 2A, filled circles with solid line).

The effects of subcellular Na^+^ channel distribution on CV differed between in the cleft and the conventional non-cleft models (Figures 2A and 2B). In both models, the peak *I*_Na,LM_ of each myocyte was proportional to the %*g*_Na,LM_. However, in the cleft model, because the small *G*_j_ at the intercellular junction (Figure 1B) attenuates the *I*_Na,JM_ that inflows into myocytes from the cleft space, the contribution of *I*_Na,LM_ to the total peak *I*_Na_ per myocyte was larger than that of *I*_Na,JM_ (Figure 2C). The decrease in *I*_Na,LM_, which accounts for a large proportion of the total *I*_Na_, by reducing %*g*_Na,LM_ while maintaining %*g*_Na,JM_ led to a marked decrease in the total inward Na^+^ current within a myocyte (Figure 2C, *left*). Since then, the intracellular Na^+^ concentration near the pre-JM (near the cleft) becomes lower, the *I*_Na,JM_ flow from the cleft to the myocyte via the pre-JM slightly increases (Figure 2C, *left*, magenta bars) and therefore the cleft voltage polarizes more negatively, resulting in the faster depolarization in the post-JM of the adjacent myocyte (i.e., EF mechanism) [16]. Because the gap junctional current between the myocytes is very large and is determined by the difference in membrane potentials between pre- and post-JMs, the faster depolarization in the post-JM (smaller time delay) via the EF mechanism largely decreases the gap junctional current. Thus, the axial current flow within the adjacent myocyte markedly decreases and the LM of the adjacent myocyte depolarizes slower, resulting in the conduction delay. Therefore, reducing %*g*_Na,LM_ resulted in a significant decrease in CV (Figure 2A). This dependence on the %*g*_Na,LM_ of the CV in the cleft model was also confirmed by additional simulations (Figure S2A, horizontal contour lines), suggesting %*g*_Na,LM_ rather than %*g*_Na,JM+LM_ is the main determinant of CV.

In contrast, in the conventional non-cleft model, which did not consider the *G*_j_ (Figure 1C), there was no difference in the contribution of *I*_Na,JM_ or *I*_Na,LM_ to the total peak *I*_Na_ of each myocyte (Figures 2D, *left* and *right*). Therefore, %*g*_Na,JM+LM_ (i.e., the total number of Na^+^ channels per myocyte) rather than %*g*_Na,LM_ (i.e., the heterogeneous distribution of Na^+^ channels within each myocyte) was the predominant determinant of CV in the non-cleft model (Figure 2B). This was further confirmed by the vertical contour lines of CV in the non-cleft model (Figure S2B). Although this result is concordant with those of the previous study [32], even with 0%*g*_Na,LM_, the conventional non-cleft model was unable to reproduce the conduction slowing [9] caused by the subcellular Na^+^ channel redistribution in the IBZ [14] (Figure 2B, filled circles with solid line).

### Contribution of a cleft at the lateral membrane to the EF mechanism

As the interstitial cleft space between adjacent myofibers is visible by optical microscopy, the width of interstitial space (facing at lateral membrane) is much larger than that of the intercellular cleft space facing at the IDs (∼1 µm vs. 5–25 nm). Previous studies [12], [16] found that when the intercellular cleft width is above 50 nm, the effect of the EF mechanism on excitation conduction is lost. Therefore, the effects of EF mechanism via clefts at the lateral membrane on the excitation conduction can be ignored.

### Contribution of gap junctions to conduction slowing in the IBZ

Acute myocardial ischemia-mediated gap junction lateralization decreases the *G*_g_ of IDs, resulting in conduction slowing [33]–[35]. In the present study, we assumed the *G*_g_ in the IBZ was equal to that in the NZ [8], [9]. Nevertheless, the aforementioned experimental data [9] could be reproduced only by reducing %*g*_Na,LM_ without altering *G*_g_ in the cleft model (Figure 2A). Therefore, we consider that the conduction slowing and induction of reentrant tachyarrhythmia in the IBZ are due to the subcellular Na^+^ channel redistribution rather than the decrease in *G*_g_. This notion is supported by a recent experimental study [36] showing that the Na^+^ channel expressions of the LM in dystrophin-deficient mouse hearts are selectively decreased without affecting the connexin 43 expressions; consequently, the CV in the left ventricle was 28% slower in the mutant mice than the wild type.

### Proarrhythmic effects of class I antiarrhythmic drugs under ischemia

Excitation conduction was more easily blocked by Na^+^ channel blockade in the IBZ than the NZ because of the marked decrease in the excitability of IBZ (Figure 3A). Accordingly, we speculate that subcellular Na^+^ channel redistribution together with Na^+^ channel blockade causes a unidirectional block at sites near the IBZ. In addition, the CV restitution curves under Na^+^ channel blockade in the IBZ (Figure 4B) suggest that PVCs can easily causes the unidirectional block, leading to reentry in old myocardial infarction patients treated with class I antiarrhythmic drugs. Indeed, Na^+^ channel blockade with the coexistence of an NZ and IBZ widened the vulnerable period for PVCs (Figure 5A, red region) compared to the cases with <14%*G*_Na_ block. Furthermore, unidirectional block induced by a stimulus applied at a site near the IBZ initiated reentry (Figure 5B(c)).

On the other hand, as shown in Figure 3A, there existed a range of Na^+^ channel blockade causing unstable conductions (Figures 3C and 3D) between the ranges of stable conduction (Figure 3B) and conduction block (Figure 3E). Such unstable conductions might be involved in arrhythmogenic mechanisms under ischemia [37], [38]. Baba *et al*. [14] have reported that the subcellular Na^+^ channel redistribution occurs inhomogeneously at the IBZ [14]. Therefore, spatially inhomogeneous Na^+^ channel blockade also occurs in the IBZ, causing unstable conduction. Taken together, the present results suggest that even if the number of PVCs is reduced as a result of the continuous administration of class I antiarrhythmic drugs, the subcellular Na^+^ channel redistribution under Na^+^ channel blockade increases the ventricular vulnerability to PVCs, leading to the initiation of reentrant tachyarrhythmias and consequently more arrhythmic events [1], [2].

### Limitations

We did not take account the ischemia-related electrical remodeling of Na^+^ channels [3], [4]. Nevertheless, as demonstrated in Figure 2C, *I*_Na_ can be decreased *only* by subcellular Na^+^ channel redistribution. Moreover, we considered neither the other electrical remodelings in the IBZ [5]–[7] nor a realistic ventricle shape. Therefore, additional studies are required to clarify the precise roles of the remodeling of other ion channels as well as the more sophisticated 2- and 3-dimensional ventricular models [39] in the ischemia-related proarrhythmic effects of class I antiarrhythmic drugs.

## Supporting Information

Figure S1
**Equivalent circuit of the cleft models.** Each membrane segment comprises a modified Luo–Rudy dynamic (mLRd) model and membrane capacitance, *C*_m,*k*_, for *k* = 1, 2, and 3. 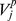 denotes extracellular cleft potential just after the *p*th myocyte. The values 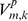, 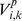, and 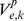 represent the transmembrane, intracellular, and extracellular potentials, respectively, of *k*th segment of the *p*th myocyte. The values *k* = 1 and 3 denote junctional membrane (JM) segments, and 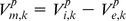.(EPS)

Figure S2 **Conduction velocity versus subcellular Na^+^ channel distributions in each myofiber model.** (A) and (B), Conduction velocity (CV) in the cleft (A) and non-cleft (B) models as functions of %*g*_Na,JM+LM_ and %*g*_Na,LM_. The contour intervals are 10 cm/s.
(EPS)

Figure S3 **Ionic mechanism of AP alternans under Na^+^ channel blockade.** AP waveforms (A) and Na^+^ channel currents (B), and L-type Ca^2+^ channel currents (C) observed in the LM segment of the 150th cell in the NZ and IBZ myofibers with 50%*G*_Na_ block, respectively. (B), Inset shows an enlarged diagram of the time course in Na^+^ channel current in the IBZ myofiber.
(EPS)

Figure S4 **Ionic mechanism of reflection observed in the myocardial ring model.** AP waveforms (A), gap junctional currents (B), Na^+^ channel currents (C), and L-type Ca^2+^ channel currents (D) observed in myocytes located at the near border between the NZ and IBZ of the myocardial ring model with 50%*G*_Na_ block. (A), Solid and dashed lines with arrowheads indicate counterclockwise rotating conduction and a clockwise rotating conduction, respectively. (B), Gap junctional current that flows into the (*p*−1)th myocyte from the *p*th myocyte was defined as negative current. (C), The inset shows an enlarged diagram of the time course in Na^+^ channel currents.
(EPS)

Text S1 **Expanded Methods.**
(DOC)
