# Supplementary material for: Ischemia-Related Subcellular Redistribution of Sodium Channels Enhances the Proarrhythmic Effect of Class I Antiarrhythmic Drugs: A Simulation Study
Source: PLoS One. 2014 Oct 3;9(10):e109271. doi: 10.1371/journal.pone.0109271 (PMC4184874; doi:10.1371/journal.pone.0109271)
Supplement: Text S1 — Expanded Methods. (DOC) [file pone.0109271.s005.doc]

**Supporting Material**

**Text S1**

**Ischemia-related Subcellular Redistribution of Sodium Channels Enhances the Proarrhythmic Effect of Class I Antiarrhythmic Drugs: A Simulation Study**

Kunichika Tsumoto, Takashi Ashihara, Ryo Haraguchi, Kazuo Nakazawa, and Yoshihisa Kurachi

**Expanded Methods**

***Myocardial fiber and ring models***

First, we constructed a myofiber model comprising 300 ventricular myocytes (Figures 1A and S1). As with our previous study [16], the cytosolic conductance (*G*_i_) of each myocyte was 3.584 μS, calculated from *G*_i_ = σ_myo_∙π∙*r*^2^/*l*, where σ_myo_ (6.7 mS/cm) is the cytosolic conductivity [12, 21], and *l* ($100\sqrt{2}$μm) and *r* ($11\sqrt{2}$μm) are the length of the myocyte and radius of the myocyte, respectively [18-20]. In addition, the radial cleft conductance (*G*_j_) and series axial cleft conductance (*G*_d_) were defined as *G*_j_ = 8∙π∙*w*∙σ_ext_ [22] and *G*_d_ = σ_ext_∙π∙*r*^2^/*w*, respectively, where σ_ext_ (6.7 mS/cm) is the extracellular conductivity [12, 16], and *w* (15 nm) is the cleft width, which is the distance between the pre- and post-junctional membrane (JM) [12, 16].

Second, we constructed a myocardial ring model comprising 900 ventricular myocytes (Figure 1E). The electric field mechanism together with the gap junction mechanism connected both ends of the myofiber, shown in Figure S1. Thus, the first segment of the first myocyte was connected to the third segment of the 900th myocyte.

***Ventricular myocyte model***

Each membrane segment composing the ventricular myocyte was described by a modified Luo–Rudy dynamic (mLRd) model [23-26]. The mLRd model consisted of a membrane capacitance and several ion channel currents, including some of our modifications [25, 26] to the original currents of L-type calcium channel (*I*_CaL_) and several potassium channels (*I*_Kr_, *I*_Ks_, *I*_Kp_, and *I*_to_). Furthermore, the initial values of [Na^+^]_i_ and [Na^+^]_o_ were set to 12.1 mM and 140.0 mM, respectively [24]. The mLRd model was implemented in an XML-based Physiological Hierarchy Markup Language (PHML) as an open-access resource available at the PhysioDesigner web site (http://physiodesigner.org).

***Calculations***

The calculation methods for excitation conduction in the myocardial fiber and ring models are as follows. From the equivalent circuits of the models shown in Figure S1, a current vector (**I**) is given by

$\mathbf{I}=\left[\begin{matrix} i_{m,1}^{1} & i_{m,2}^{1} & \cdots& i_{m,3}^{p-1} & i_{m,1}^{p} & i_{m,2}^{p} & i_{m,3}^{p} & i_{m,1}^{p+1} & \cdots& i_{m,2}^{N} & i_{m,3}^{N} \end{matrix}\right]^{T},$ (S1)

where [ ]*^T^* represents the transpose operation and *N* is the myocyte number. The current vector comprised the membrane current from each node toward each membrane segment. Furthermore, a voltage function vector (**V**) consisting of the functions of the transmembrane potential of each membrane segment in the *p*th myocyte is given by

$\mathbf{V}=\left[\begin{matrix} v_{1,1} & v_{1,2} & \cdots& v_{p-1,3} & v_{p,1} & v_{p,2} & v_{p,3} & v_{p+1,1} & \cdots& v_{N,2} & v_{N,3} \end{matrix}\right]^{T}$. (S2)

Then, each function, *v_p_*,*_k_*, for *p* = 1, …, *N* and *k* = 1, 2, and 3, except for the first function (*v*_1,1_) and the final one (*v_N_*_,3_), in the voltage vector is given by

$\begin{matrix} v_{p,1} =-k_{1} V_{m,3}^{p-1} +k_{2} V_{m,1}^{p} -V_{m,2}^{p} , \\ v_{p,2} =-V_{m,1}^{p} +2V_{m,2}^{p} -V_{m,3}^{p} , \\ v_{p,3} =-V_{m,2}^{p} +k_{2} V_{m,3}^{p} -k_{1} V_{m,1}^{p+1} , \end{matrix}$ (S3)

where *k*_1_ = 0.5*G*_g_*/G*_i_ and *k*_2_ = 1 + 0.5*G*_g_*/G*_i_. In cases of *p* = 1 and *p* = *N*, *v*_1,1_ and *v_N_*_,3_ are provided by $v_{1,1} =V_{\text{m},1}^{1} -V_{\text{m},2}^{1}$and $v_{N,3} =-V_{m,2}^{N} +V_{m,3}^{N}$, respectively. Using the current vector (Eq. S1) and the voltage vector (Eq. S2), we can derive a circuit equation at arbitrary time *t* using Ohm’s law and Kirchhoff’s law, leading to the following simultaneous equation:

$\mathbf{RI}=\mathbf{V},$ (S4)

where **R** is the 3*N* × 3*N* resistance matrix. The matrix, **R**, in Eq. S4 is given by Eq. S5:

(S5)

where *r*_0_ = 0.5/*G*_i_, *r*_1_ = 1/*G*_j_ + 0.5(1/*G*_d_ + 1/*G*_i_), *r*_2_ = 1/*G*_j_ + 0.5/*G*_d_, and *r*_3_ = 1/*G*_j_. Eq. S4 can be solved for the current ***I*** with $V_{\text{m},k}^{i}$ at time *t* as an initial condition. On the other hand, Eq. S4 in the non-cleft model can be derived by setting the cleft conductance, *G*_d_ and *G*_j_, to infinity in Eq. S5. Furthermore, in the case of the myocardial ring model, the following matrix was employed:

(S6)

The transmembrane potential in each segment is given by

$C_{m,k} \frac{\text{d}V_{m,k}^{p} }{\text{d}t}+I_{\text{ion}} \left(V_{m,k}^{p} ,t\right)=i_{m,k}^{p} ,$ (S7)

for *p* = 1, …, *N* and *k* = 1, 2, and 3, where *C*_m,k_ (μF/cm^2^) is the membrane capacitance with specific capacitance 1.0, *I*_ion_ (μA/μF) is the sum of several ion channel currents in the mLRd model, $i_{\text{m},k}^{p}$(μA/μF) is the transmembrane current corresponding to each element in the current vector defined by Eq. S1. For an arbitrary time *t*, all the membrane currents, $i_{\text{m},k}^{p}$, were obtained by solving Eq. S4 with the transmembrane potentials as an initial condition. Thus, we calculated all transmembrane potentials at time *t*+Δ*t* in each segment, where Δ*t* corresponds to the time step in the Euler method. The time step, Δ*t*, was set to 1 μs.
